# Study on the Suitable Area of Ratoon Rice in China Under Climate Change

**DOI:** 10.1002/ece3.72724

**Published:** 2025-12-17

**Authors:** Wei Luo, Lixin Yuan, Shengmin Yan, Linlin Wang, Yongfu Yu, Huan Deng, Jinpeng Zhao, Rulin Wang

**Affiliations:** ^1^ Zigong Meteorological Bureau Zigong People's Republic of China; ^2^ Zigong Academy of Agricultural Science Zigong People's Republic of China; ^3^ Environment‐Friendly and Efficient Water‐Saving Technology and Equipment for Hilly Agriculture Key Laboratory of Sichuan Province Chengdu Sichuan People's Republic of China; ^4^ Sichuan Provincial Rural Economic Information Center Chengdu People's Republic of China

**Keywords:** climate change, MaxEnt model, ratoon rice, suitable area

## Abstract

Ratoon rice is a special cultivation system developed based on the regenerative capacity of rice axillary buds. Its core mechanism lies in regulating the regeneration potential of stubble axillary buds through agronomic practices. After harvesting the main‐season rice, dormant buds are induced to sprout and form secondary tillers, which eventually develop into effective panicles, thereby achieving “one planting with two harvests.” In this study, 167 occurrence records of ratoon rice, nine environmental variables, and three future climate scenarios proposed in CMIP6 (SSP1‐2.6, SSP2‐4.5, and SSP5‐8.5) were used to predict the suitable cultivation area of ratoon rice in China using the MaxEnt model. The results showed that the main environmental factors influencing the distribution of ratoon rice were accumulated temperature during the safe growth period, length of the safe growth period, altitude, hydrothermal coefficient in August, mean temperature in September, and precipitation from March to September. The optimal ranges of these factors were 4314°C–5497°C, 191–225 days, 0–511 m, 0.3–11.4, and 21.0°C–25.2°C, respectively. Under the current climate scenario, the potential suitable area of ratoon rice in China was mainly distributed south of the Qinling–Huaihe line (92.39°–121.96° E, 18.23°–30.36° N), covering a total area of 193.90 × 10^4^ km^2^. Compared with the current scenario, the total suitable area of ratoon rice is projected to increase by 5.5%–11.9% in the 2050s and by 8.5%–9.6% in the 2090s under the three climate scenarios. By the 2050s, the suitable and highly suitable areas in Sichuan and Chongqing show little change across the three climate scenarios, whereas the suitable areas in the middle and lower reaches of the Yangtze River increase markedly. The newly expanded suitable areas are mainly concentrated in southern Hunan, the central parts of Hubei, Jiangxi, and Anhui, and western Zhejiang. By the 2090s, the suitable and highly suitable areas in Sichuan and Chongqing still exhibit little change under the three climate scenarios, while the highly suitable areas in the middle and lower reaches of the Yangtze River continue to expand, with newly added highly suitable zones comparable to those in the 2050s. Accordingly, we infer that autumn thermal conditions north of the Qinling–Huaihe line will remain a limiting factor for the northward expansion of ratoon rice, whereas moderately increased autumn temperatures south of the Qinling–Huaihe line will be favorable for ratoon rice cultivation. Most moderately and marginally suitable zones in the middle and lower reaches of the Yangtze River are expected to shift into highly or moderately suitable zones, especially in southern Hunan, the central parts of Hubei, Jiangxi and Anhui, and western Zhejiang, where new highly suitable areas are projected to emerge. These regions could therefore be prioritized for appropriately scaled ratoon rice cultivation in the future.

## Graphical abstract

In view of this, we speculate that the autumn heat conditions in the northern Qinling–Huaihe Line will still limit the northward advance of ratoon rice. With future climate change, a small part of the highly‐suitable area for ratoon rice in the southern Qinling–Huaihe Line will transform into a double‐cropping rice area, while most of the lowly‐suitable areas of ratoon rice in the middle and lower reaches of the Yangtze River will be transformed into moderately‐suitable areas and above. Southern Hunan, Hubei, Jiangxi, central Anhui, and western Zhejiang were newly added highly‐suitable areas, and large‐scale ratoon rice cultivation can be carried out in the future.

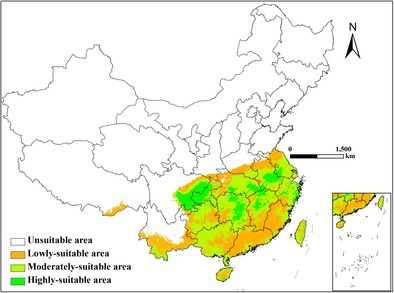

## 1 Introduction

Ratoon rice is a unique rice planting mode, which refers to the production of a second rice crop from the stubble left behind after the harvest of the main crop (Yuan et al. 2019). Rice ratooning has been adopted in many countries, such as China, India, Japan, the USA, the Philippines, Brazil, Colombia, Swaziland, and Thailand (Golam et al. 2014). It has the advantages of saving labor, saving seed, saving water, saving fertilizer, saving medicine, and so on, and the rice quality is excellent (Wang, He, et al. 2020; Wang, Feng, et al. 2020). Therefore, rice ratooning is an important measure and effective way to make full use of autumn climate resources to improve the multiple cropping index, increase yield per unit area of rice field, and ensure food security in southern China where heat resources are “insufficient in two seasons and excess in one season” (Li et al. 2022). According to statistics, more than 1.2 million hectares of ratoon rice are planted in 11 provinces and cities, such as Fujian, Jiangxi, Zhejiang, Hunan, Hubei, Anhui, Jiangsu, Sichuan, Chongqing, Yunnan, and Guizhou. It is estimated that China's potential ratoon rice planting area is more than 5 million hectares, and it can increase production by 20–30 billion kilos per year according to the regeneration season yield of 4000 to 6000 kg·hm^−2^ (Lin et al. 2024). Therefore, ratoon rice has become a rice planting model with remarkable productivity in China at present.

The research on ratoon rice is very extensive, including high‐yield cultivation techniques (Xu et al. 2021; Hu et al. 2023; Xie 2017), planting influencing factors (Song et al. 2020; Xie et al. 2016; Zheng et al. 2022), planting division (He et al. 2013), etc. In terms of agricultural climate division, there are more studies on current conditions, but few studies on the impact of future climate change on ratoon rice. Tu (2006) takes the safe growing season of rice as the index and adopts the climate analysis method based on grid points to divide rice planting region in Guangxi into four climate suitable regions, namely the late‐maturing double‐crop rice region, the middle and late‐maturing double‐crop rice region, the early and middle‐maturing double‐crop rice region, and the single‐crop ratoon rice region. According to the relationship between the growth, development and yield formation of ratoon rice in southeastern Sichuan Basin and meteorological conditions, combined with the characteristics of climate resources in Chongqing, Gao et al. (2011) calculated the spatial distribution of regionalization indicators by using ARCGIS software and carried out a refined regionalization about the suitability of photothermal resources for ratoon rice in Chongqing. Wang (2013) analyzed the change trend of rice yield, area and climate factors in Guangxi in the past 50 years, explored the main climate factors affecting rice yield, and carried out climate regionalization of rice planting. In recent years, with the intensification of global warming, the living environment, genetic breeding, distribution pattern and traits of species have been greatly affected (Guo 2015; Yanagi 2024; Kelemu et al. 2023), which will inevitably affect the planting of ratoon rice. Therefore, it is of positive significance to study the habitat changes and character evolution of ratoon rice under climate change in advance for coping with the climate change risks and adjusting the industrial structure and layout of rice.

CMIP6 is a new phase of the Coupled Model Alignment project launched to address new scientific questions in the field of climate change and provide data to support the scientific objectives established by the World Climate Research Program (WCRP) (Zhou et al. 2019). According to research of Deng (Deng 2021), the performance of the eight Earth system models on China's temperature simulation ability considering the time and spatial scale in order from high to low is ESM2‐WACCM, CAMS‐CSM1‐0, CESM2, CNRM‐CM6‐1, BCC‐ESM1, BCC‐CSM2‐MR, CanESM5, CNRM‐CM6‐1. Therefore, we choose the ESM2‐WACCM model as the future climate change system model.

In 2025, the Ministry of Agriculture and Rural Affairs of China issued the *Guidelines for Key Tasks to Promote the Development of Ratoon Rice* (*2025–2030*), which set the goal of expanding the national ratoon rice cultivation area by approximately 0.67 × 10^4^ km^2^ by 2030. Given China's complex and diverse topography and climate, how to effectively utilize regional climatic resources under the context of global climate change and ensure that the expansion of ratoon rice cultivation is well aligned with available climate resources has become an urgent issue. At present, most studies on the climatic suitability of ratoon rice focus on individual provinces, while research addressing the spatiotemporal dynamics of ratoon rice cultivation under future climate change scenarios across China remains limited. Moreover, the potential distribution of ratoon rice suitability zones under projected climate scenarios is still unclear. To address these gaps, this study employed the MaxEnt model in combination with ArcGIS spatial analysis techniques to predict the impacts of climate change on the cultivation patterns and characteristics of ratoon rice in China. Specifically, this study aims to answer the following questions: (1) What are the distributional characteristics of ratoon rice cultivation in China under climate change? (2) How do different climatic factors influence the spatial distribution of ratoon rice? Answering these questions will contribute to understanding the drivers of geographical distribution shifts of ratoon rice and provide scientific support for achieving the strategic goal of expanding the ratoon rice area by 0.67 × 10^4^ km^2^ in China by 2030 under climate change.

## 2 Materials and Methods

### 2.1 Data Sources and Processing

The base map of China was obtained from the National Geospatial Information Service Platform (https://www.tianditu.gov.cn/), with map approval number GS (2024) 0650. Meteorological data from January 1, 2000, to December 31, 2020, were provided by the National Meteorological Information Center, including daily mean surface temperature and daily mean precipitation. Stations with more than 30 consecutive missing records were excluded, and missing values at the remaining stations were supplemented using spline interpolation written in Python. Soil data were derived from the Harmonized World Soil Database (HWSD, V1.2), including soil pH and soil organic matter (SOM). The Human Footprint Index (Hf) was obtained from the Center for International Earth Science Information Network (CIESIN).

Future climate data were obtained from the latest CMIP6 dataset (aims2.llnl.gov). Simulations were conducted using the BCC‐CSM2‐MR global climate model based on CMIP6, which improves both horizontal and vertical resolution for enhanced simulation accuracy. Future climate scenario data covering the period from January 1, 2041, to December 31, 2080, were downloaded from the Earth System Grid Federation (ESGF, https://aims2.llnl.gov/search). Three scenarios were selected: SSP1‐2.6, SSP2‐4.5, and SSP5‐8.5. Among them, SSP1‐2.6 represents a low‐emission sustainable development pathway in international climate research, SSP2‐4.5 reflects a relatively likely scenario for most countries pursuing sustainable development, while SSP5‐8.5 represents a high‐emission pathway with potentially severe consequences. Model data were resampled to a 30‐arc second resolution using bilinear interpolation.

**FIGURE 1:**
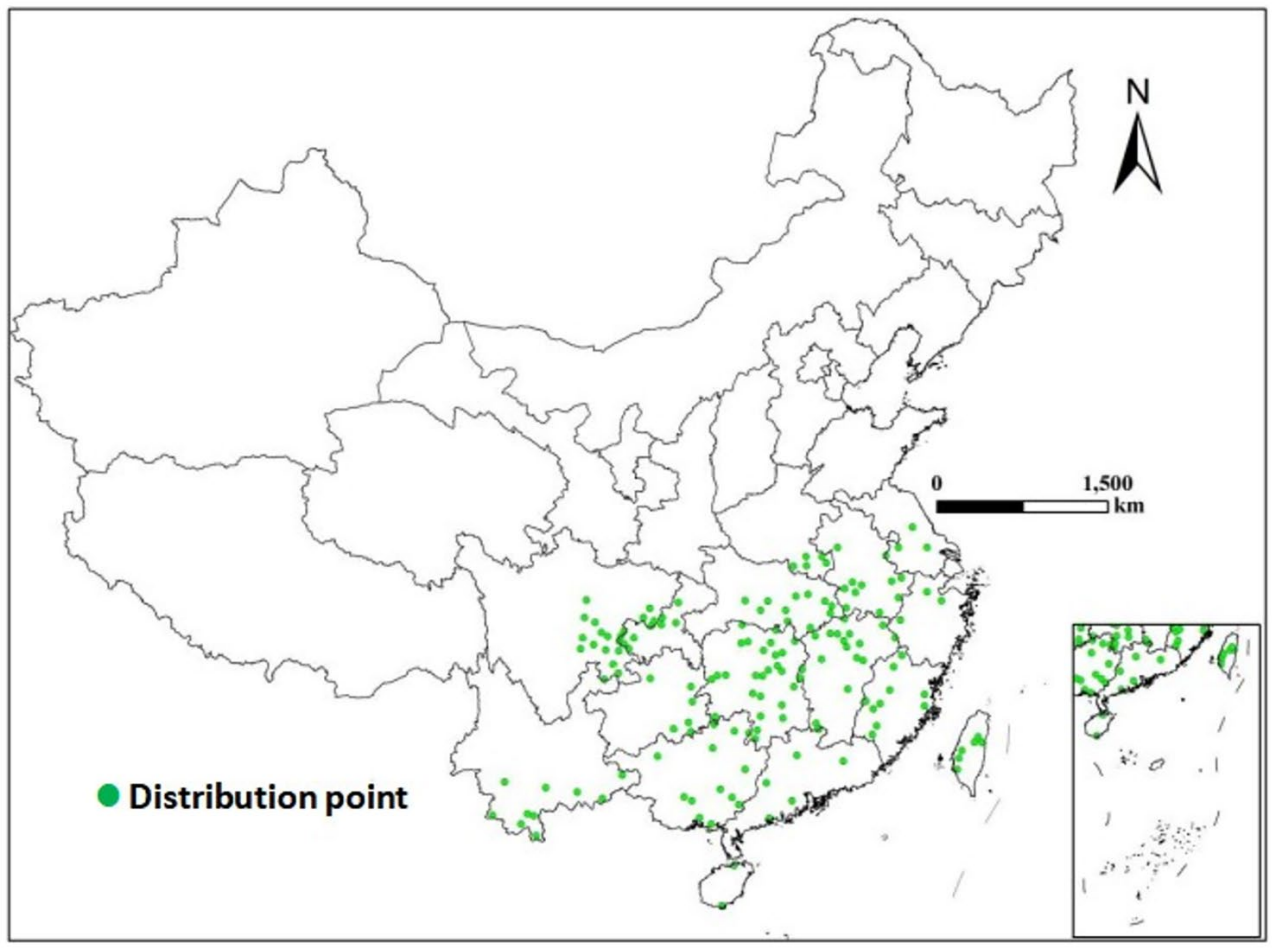
Geographical distribution records of ratoon rice in China.

Distribution records of ratoon rice were obtained from the Institute of Rice and Sorghum, field surveys, and published literature (Tang et al. 2020; Xu et al. 2023; Yao et al. 2013; He et al. 2024; Song et al. 2020). For records lacking latitude and longitude information, geographic coordinates were retrieved using Google Earth. The distribution data were further filtered using ENMTOOL software to remove duplicate records within the same 30‐arc second grid cell, thereby avoiding spatial autocorrelation caused by multiple records in a single grid. After data processing, 175 records were retained for species distribution modeling, and the specific distribution points are shown in Figure 1.

Based on the existing research on the planting regionalization of ratoon rice, the climatic factors affecting the growth of ratoon rice (He et al. 2006; Fang et al. 1994; Sichuan Meteorological Bureau Agricultural Meteorological Center Ratoon Rice Research Group 1990), and the characteristics of current growing areas, nine potential environmental factors with clear biological significance that may affect the distribution of ratoon rice in China were selected (Table 1).

**TABLE 1:** Potential environmental factors affecting the distribution of ratoon rice in China.

Code	Environmental variable	Biological significance	Unit
T	Safe growth period accumulated temperature	Characterize the amount of heat required for safe sowing of middle‐season rice (stable through ≥ 10°C on the first day) to safe panicle filling of ratoon rice (stable through ≥ 22°C all day long).	°C
Day	Safe growth period days	Characterize the number of days required for safe sowing of middle‐season rice (stable through ≥ 10°C on the first day) to safe panicle filling of ratoon rice (stable through ≥ 22°C all day long).	day
K_8_	Hydrothermal coefficient in August	Reflecting the stress of high temperature and low humidity environment on the number of seedlings during the seedling stage of ratoon rice.	—
Tav_9_	Average temperature in September	The limitation of temperature in heading and flowering period of ratoon rice on seed setting rate.	°C
Prec_39_	Precipitation from March to September	The water supply of “medium rice + ratoon rice” during the growth process.	mm
Alt	Altitude	Effects of comprehensive environmental factors on the growth of ratoon rice at different altitudes.	m
Hf	Human footprint index	Represents the extent of human intervention in the natural environment.	—
Ph	Soil pH	Indicates the acidity or alkalinity level of soil solution.	—
Som	Soil organic matter content	Represents the fertility and health status of paddy soil.	%

*Note:* “—” indicates dimensionless.

The hydrothermal coefficient in August refers to the ratio of potential evapotranspiration to precipitation, expressed as

(1) K=r/0.1∑t

where *K* is the hydrothermal coefficient, *r* is the precipitation in August, and Σ*t* is the accumulated temperature in August. A *K* value > 1 indicates humid conditions, whereas a *K* value < 1 indicates dry conditions. This index reflects the degree of humidity in a given region.

### 2.2 Software and Parameter Settings

The software containing the MaxEnt model was downloaded from the official website (V3.4.4, https://biodiversityinformatics.amnh.org/open_source/maxent/). The accuracy of the MaxEnt model can be achieved by adjusting the regularization multiplier (RM) option and feature combinations (FCs) option. The range of RM was set to 0.5 to 4, and the step was set to 0.5. The FCs option includes linear (L), quadratic (Q), hinge (H), product (P), and threshold (T). The combination of L, LQ, H, LQH, LQHP, and LQHPT was selected in the study. Therefore, RM and FCs can form 48 different combinations, and the combinational model with the minimum Akaike information criterion correction (AICc) value was selected as the optimal model (Fu et al. 2024).

### 2.3 Model Accuracy Evaluation Method

The area under receiver operating characteristic curve (AUC) is an important indicator to measure the model performance, which has good performance. Therefore, we use the AUC value as a criterion for evaluating the model for its discrimination capacity. If the AUC value is > 0.9, the prediction effect of the model is better. If the AUC value is < 0.9 and > 0.7, the prediction effect of the model is general. If the AUC value is < 0.7, the prediction effect of the model is poor.

### 2.4 Suitable Area Division

The calculation results of MaxEnt were loaded in ArcGIS 10.2, and the suitable biological grade was divided and visualized to obtain the potential distribution map of species (The projection coordinate system was GCS_WGS_1984). According to the research of Sun et al. (2012), the probability of species existence was divided into four levels, namely the unsuitable area (the probability of occurrence < 0.05), the lowly‐suitable area (the probability of occurrence is 0.05~0.33), the moderately‐suitable area (the probability of occurrence is 0.33~0.66), and the highly‐suitable area (the probability of occurrence more than 0.66). The changes of the potential distribution area of ratoon rice were mainly calculated using the SDM toolbox (Etherington 2011), which was mainly written based on python.

## 3 Results

### 3.1 Importance of Environmental Factors

**FIGURE 2:**
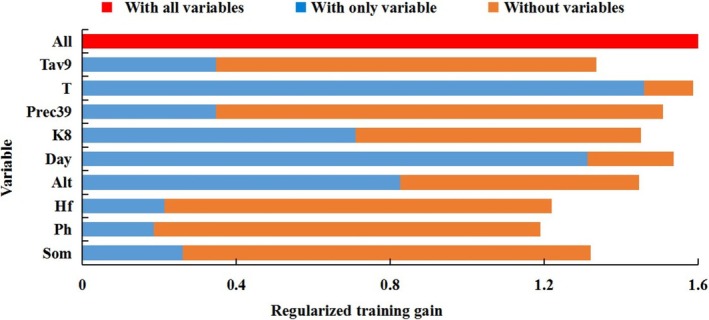
The importance of environmental factors in Jackknife test.

The importance of each environmental variable in the simulation was evaluated using the jackknife test (Figure 2). Among them, the training gains of accumulated temperature during the safe growth period (T) and duration of the safe growth period (Day) ranged from 1.3 to 1.5, contributing the most to the distribution of ratoon rice, indicating that these two variables are the most critical environmental factors. Elevation (Alt) and the hydrothermal coefficient in August (K_8_) had training gains between 0.7 and 0.9, also playing an important role in the distribution of ratoon rice. The mean temperature in September (Tav_9_) and precipitation from March to September (Prec_39_) had training gains between 0.3 and 0.4, suggesting a certain degree of influence. In contrast, the human activity index (Hf), soil pH (Ph), and soil organic matter content (Som) had training gains between 0.2 and 0.3, indicating relatively minor effects on the distribution of ratoon rice.

### 3.2 Response to Key Environment Variable

**FIGURE 3:**
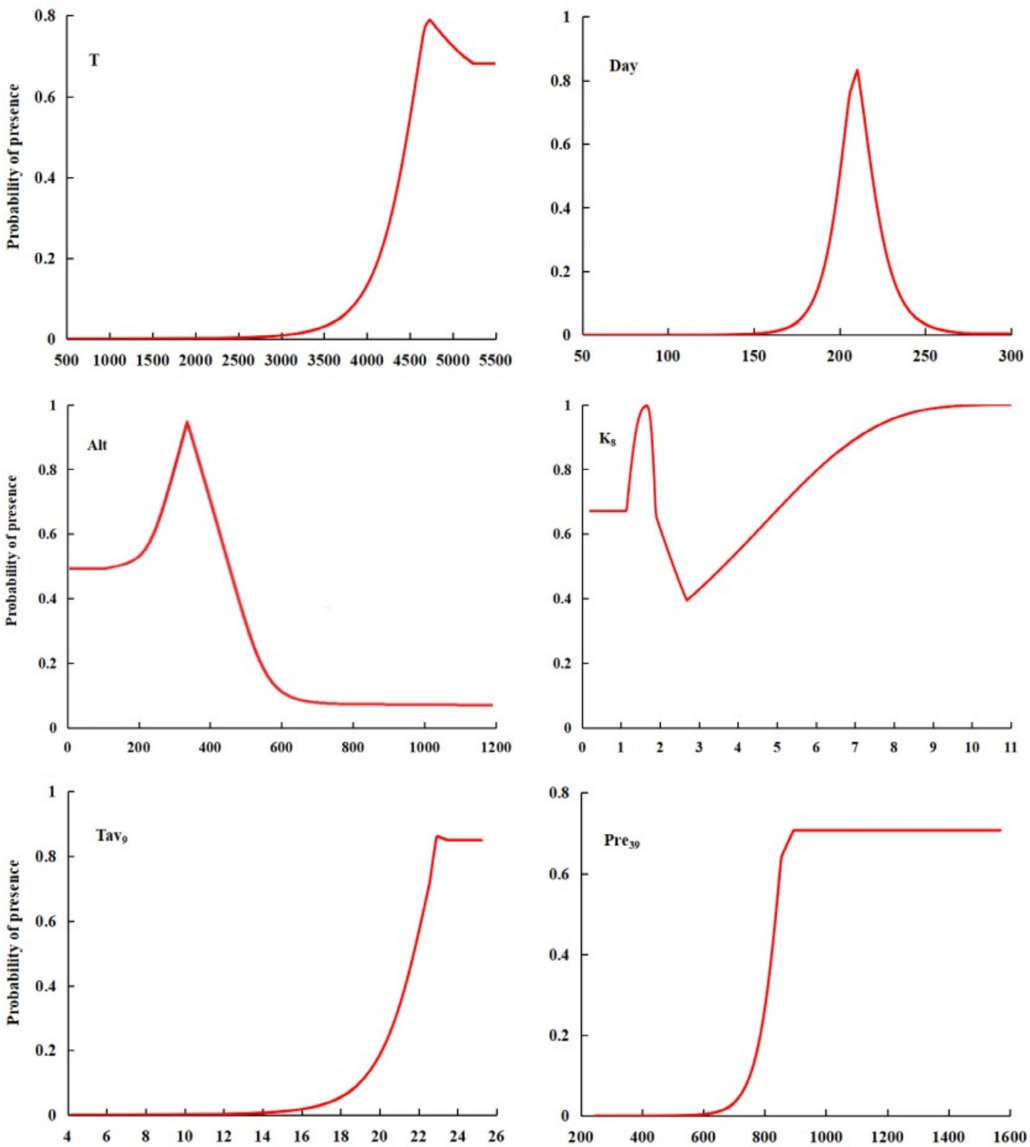
Response curves of presence probability of ratoon rice to important environmental factors.

To clarify the relationship between dominant environmental factors and the probability of ratoon rice occurrence, response curves of dominant environmental variables against distribution probability were generated using the MaxEnt model, reflecting the value ranges of environmental variables under different thresholds. Following the classification method of relevant scholars, a distribution probability of 0.33 was adopted as the threshold to determine the suitable ranges of dominant environmental variables for ratoon rice. The results (Figure 3) showed that the suitable ranges for accumulated temperature during the safe growth period, duration of the safe growth period, elevation, hydrothermal coefficient in August, mean temperature in September, and precipitation from March to September were 4314°C–5497°C, 191–225 days, 0–511 m, 0.3–11.4, 21.0°C–25.2°C, and 885–1593 mm, respectively. Their optimal values were 4787°C, 211 days, 391 m, 1.7, 23.2°C, and 1325 mm, respectively.

### 3.3 Potential Suitable Area Under Current Scenario

**FIGURE 4:**
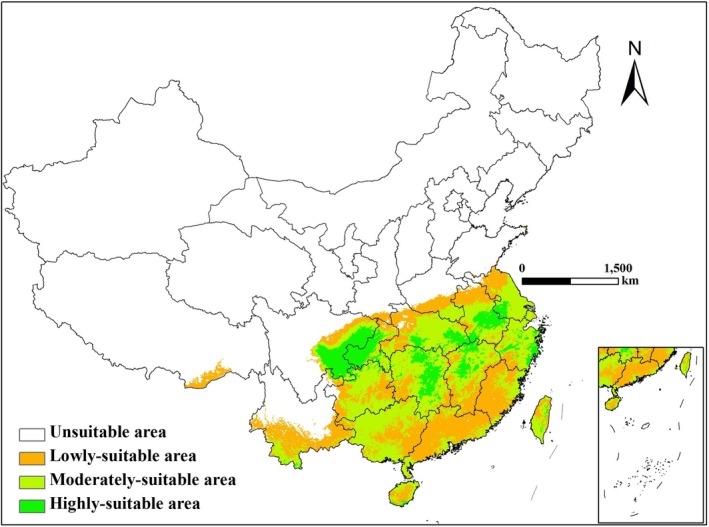
Potential suitable areas of ratoon rice under current scenario in China.

According to Figure 4 and Table 2, under the current climate scenario, the potential suitable distribution area of ratoon rice in China is mainly located south of the Qinling–Huaihe Line (92.39°–121.96° E, 18.23°–30.36° N), with its northern boundary at 30° ± 1° N. The total area reaches 193.90 × 10^4^ km^2^, accounting for 20.19% of China's land area. Highly suitable areas for ratoon rice are primarily distributed in the Sichuan Basin, western Chongqing, southern and eastern Hubei, central and eastern Hunan, north‐central Jiangxi, and central Anhui and Taiwan (103.22°–117.09° E, 26.39°–29.01° N), covering an area of 28.02 × 10^4^ km^2^. Among them, provinces with relatively large highly suitable areas include Sichuan (7.01 × 10^4^ km^2^), Hunan (4.59 × 10^4^ km^2^), Hubei (4.18 × 10^4^ km^2^), and Chongqing (2.44 × 10^4^ km^2^). Moderately suitable areas are distributed around the highly suitable regions, mainly in Guizhou, Guangxi, Anhui, Zhejiang, most of Taiwan, southern Hunan and Jiangsu, central Hubei and Jiangxi, Hainan, and eastern Fujian, covering 88.06 × 10^4^ km^2^ and accounting for 45.42% of the total suitable area. Low‐suitability areas are distributed south of the Qinling–Huaihe Line, including Tibet, Yunnan, southern Jiangxi, most of Guangdong, Hainan, and western Fujian, with a total area of 77.82 × 10^4^ km^2^, representing 40.13% of the total suitable area. The actual occurrence points of ratoon rice almost completely overlap with these suitable regions, indicating that its observed distribution (Figure 1) is largely consistent with the simulation results.

**TABLE 2:** Suitable regions of ratoon rice under different climate scenarios in China.

Scenario	Age	Total area (A/km^2^)	Highly‐suitable area (A/km^2^)	Moderately‐suitable area (A/km^2^)	Lowly‐suitable area (A/km^2^)
Current	Current	193.90	28.02	88.06	77.82
SSP1‐2.6	2050s	204.57	41.94	102.20	60.43
	2090s	210.33	37.92	108.94	63.47
SSP2‐4.5	2050s	217.08	44.48	96.99	75.61
	2090s	212.50	45.77	104.45	62.28
SSP5‐8.5	2050s	207.66	42.13	98.84	66.69
	2090s	211.39	50.62	100.06	60.71

### 3.4 Potential Suitable Area Under Future Scenarios

Compared with the current climate (Table 2), the total suitable area of ratoon rice shows an increasing trend under all three future climate scenarios, with increases of 5.5%–11.9% in the 2050s and 8.5%–9.6% in the 2090s. The highly suitable area increases substantially, with increments of 49.7%–58.7% in the 2050s and 35.3%–80.7% in the 2090s. The moderately suitable area also increases, with increments of 10.1%–16.1% in the 2050s and 13.6%–23.7% in the 2090s. In contrast, the low‐suitability area decreases under the three future climate scenarios, with reductions of −22.3% to −2.8% in the 2050s and −22.0% to −18.4% in the 2090s. Overall, the results indicate that under all three future climate scenarios, both the total suitable area and the moderately suitable area of ratoon rice will expand, the highly suitable area will increase significantly, while the low‐suitability area will shrink. The magnitude of suitable area change is greatest under the SSP2‐4.5 climate scenario, with larger changes in the 2050s compared to the 2090s.

**FIGURE 5:**
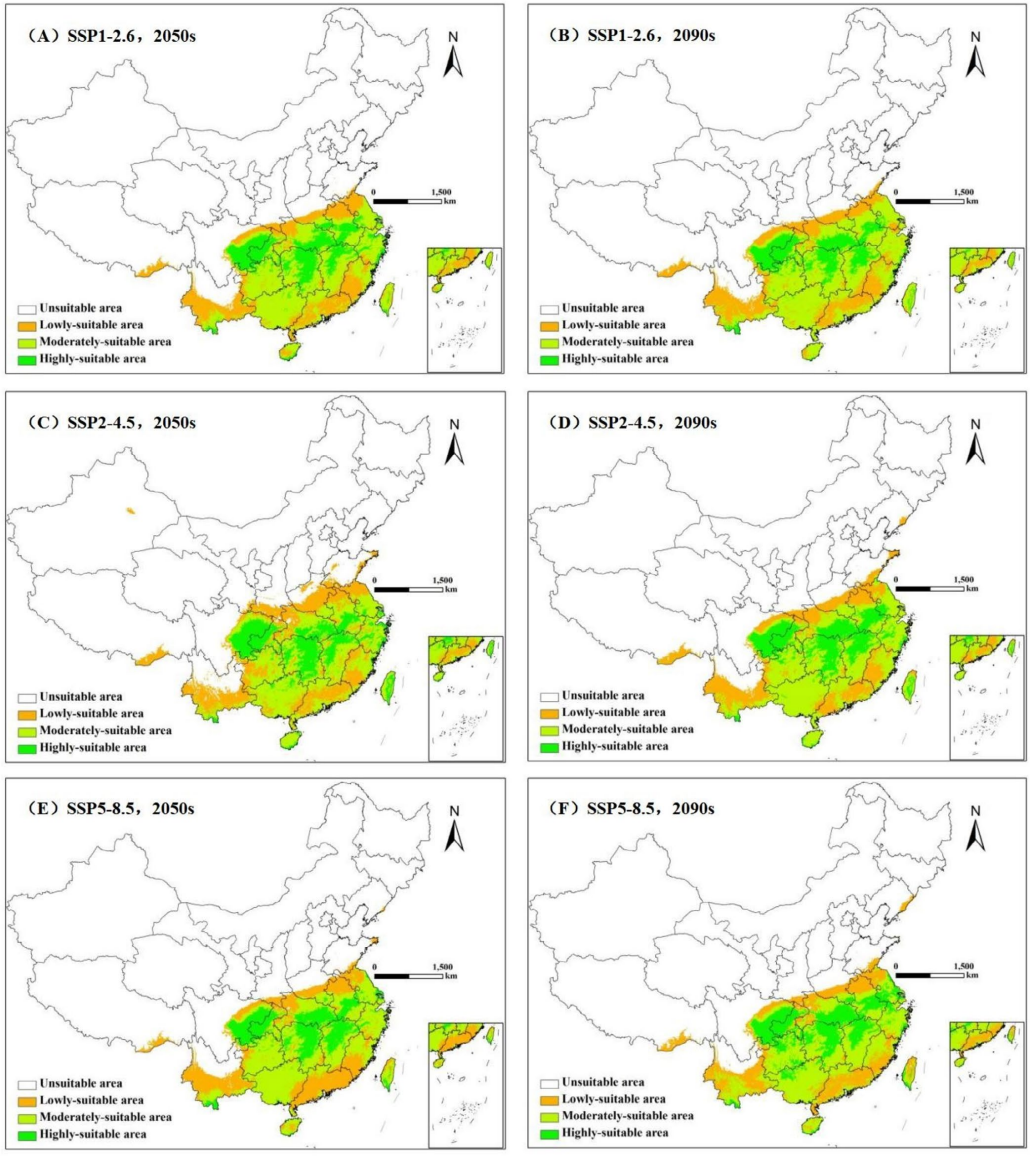
Potential suitable areas of ratoon rice under future scenarios in China. (A–F represent different climate scenarios and time periods.)

Although the area of highly‐suitable, moderately‐suitable and lowly‐suitable areas of ratoon rice are different under the three scenarios, the suitable areas are still mainly distributed in the southern Qinling–Huaihe line (Figure 5 and Table 2). By the 2050s, the area of total suitable areas and highly‐suitable areas in Sichuan and Chongqing have little change under three climate scenarios, while the area of highly‐suitable areas in the middle and lower reaches of the Yangtze River show significant increase, and the new highly‐suitable areas are mainly in southern Hunan, the central parts of Hubei, Jiangxi, and Anhui, and western Zhejiang. More specifically, the moderately‐suitable areas in southern Henan and southern Hainan will transform into highly‐suitable areas under the SSP1‐2.6 scenario, and most of lowly‐suitable areas in Guangxi will transform into moderately‐suitable areas. The boundary of the suitable areas will extend northward under the SSP2‐4.5 scenario, and the area of highly‐suitable areas in China will reach the largest, accounting for 20.5% of the total suitable area. Meanwhile, the area of highly‐suitable areas will decrease significantly in Henan, while the area of moderately‐suitable and highly‐suitable areas will increase in Hainan and Taiwan. The boundary of the suitable areas shrank slightly to the south under the SSP5‐8.5 scenario, but the lowly‐suitable areas in central Guizhou, western Guangxi and southern Fujian will turn into moderately‐suitable areas.

By the 2090s, the area of suitable areas and highly‐suitable areas of ratoon rice in Sichuan and Chongqing still have little change under three climate scenarios. Meanwhile, the area of highly‐suitable areas in the middle and lower reaches of the Yangtze River still show significant increase, and the new highly‐suitable areas will be the same as in the 2050s. More specifically, the area of lowly‐suitable areas will reach the maximum under SSP1‐2.6 scenario, accounting for 30.2% of the total suitable area. The low‐suitable areas in eastern Guangxi and central Hainan will transform into moderately‐suitable areas under SSP2‐4.5 scenario. Under the SSP5‐8.5 climate scenario, the lowly‐suitable areas in southern Yunnan will become moderately‐suitable areas, the moderately‐suitable areas in northern Guizhou and northern Guangxi will become highly‐suitable areas, the moderately‐suitable areas in central Taiwan will become lowly‐suitable areas, and the area of lowly‐suitable areas in southeastern Tibet will be significantly reduced. In general, the viability of ratoon rice will be significantly improved under the future climate change scenario, especially in the middle and lower reaches of the Yangtze River in China. Therefore, we can know that the future climate conditions will be very suitable for the planting and cultivation of ratoon rice, and appropriate large‐scale planting can be carried out in this region.

### 3.5 Centroid Movement Trajectory of Highly‐Suitabie Areas

According to the method of Zhao et al. (2023), the change of centroid movement trajectory of highly‐suitable areas under different scenarios was calculated (Table 3). The geometric center of the high‐suitability areas under the current scenarios is located in Yongshun, Hunan. For the future, the geometric center will move 149.85 km to Linli under the SSP1‐2.6 scenario, will move 138.05 km to Taoyuan, Hunan under the SSP2‐4.5 scenario, and will move 114.22 km to Taoyuan, Hunan under the SSP5‐8.5 scenario.

**TABLE 3:** Centroid movement trajectory of highly‐suitable area of ratoon rice under climate change scenarios.

Scenario	Period	Angle/°	Direction	Displacement/km
SSP1‐2.6	From current to 2050s	2.75	Northeast	113.61
	From 2050s to 2090s	23.81	Northeast	40.26
	From current to 2090s	8.52	Northeast	149.85
SSP2‐4.5	From current to 2050s	349.11	Southeast	125.02
	From 2050s to 2090s	63.03	Northeast	30.78
	From current to 2090s	4.19	Northwest	138.05
SSP5‐8.5	From current to 2050s	1.51	Northeast	104.85
	From 2050s to 2090s	70.73	Northeast	20.66
	From current to 2090s	11.34	Northeast	114.22

*Note:* The angles in the table, start from north and the calculation is clockwise.

## 4 Discussion

The growth and development of ratoon rice are closely related to climatic conditions (Xu et al. 2020; Luo, Mei, et al. 2024; Luo, Fang, et al. 2024; Duan et al. 2021). Based on previous studies on the climatic suitability of ratoon rice and combined with the MaxEnt model, this study identified six dominant environmental variables influencing the distribution of ratoon rice. Their relative importance was ranked as follows: accumulated temperature during the safe growth period, duration of the safe growth period, elevation, hydrothermal coefficient in August, mean temperature in September, and precipitation from March to September. Among these, accumulated temperature and duration of the safe growth period contributed the most to the distribution of ratoon rice, with suitable ranges of 4314°C–5497°C and 191–225 days, respectively. A study on the climatically suitable planting areas of ratoon rice in the Sichuan Basin reported that the suitable ranges of accumulated temperature and duration of the safe growth period were ≥ 4300°C and ≥ 180 days, which are consistent with our findings (Sichuan Meteorological Bureau Agricultural Meteorological Center Ratoon Rice Research Group 1990). In addition, a study on the planting layout of double‐cropping rice in Guangxi under climate change found that the suitable ranges of accumulated temperature and duration of the safe growth period within the growing season were ≥ 5600°C and ≥ 225 days (Huang et al. 2020). These studies suggest that insufficient accumulated temperature and shorter duration of the safe growth period would cause suitable areas for ratoon rice to shift into areas suitable for single‐cropping rice, whereas higher accumulated temperature and longer duration would shift suitable areas into those for double‐cropping rice. In summary, we boldly speculate that accumulated temperature and duration of the safe growth period restrict the expansion of ratoon rice suitable areas beyond the Qinling–Huaihe Line. These two environmental variables may be important factors underlying the concentration of highly suitable areas for ratoon rice between 26° N and 29° N.

Gao et al. (2011) found that ratoon rice in Chongqing was heading and flowering in September, and low temperature damage was easy to occur when the average daily temperature was lower than 22°C, which would lead to a decrease in seed setting rate. This result is basically consistent with the suitable range of T_9_ in our study, which ranges from 21.0°C to 25.2°C. In the future, the autumnal temperature will increase significantly (Song 2022), and it can be seen from our research that the area of highly‐suitable areas will increase significantly. Therefore, it can be inferred that the frequency and degree of low temperature in autumn will decrease significantly in the future, which may not pose a threat to the geographical distribution of ratoon rice in China. In this study, the suitable of altitude for ratoon rice ranges from 0 to 511 m, and the optimal value is 391 m. This predicted result is consistent with the actual situation, that is, the known distribution altitude of ratoon rice is 150–430 m currently (Fang et al. 1994). Similarly, when Pre_39_ ranges from 885 mm to 1593 mm, and K_8_ ranges from 0.3 to 11.4, the probability of presence of ratoon rice is high, indicating that sufficient rainfall not only replenishes the water requirement for the growth of ratoon rice, but also affects the field humidity. The growth and development of ratoon rice depends on the comprehensive effect of various environmental factors, but the response of the existence probability of ratoon rice to environmental factors follows the principle of single variable in our research. Therefore, the inferred results of this study cannot completely and accurately explain the relationship between ratoon rice and growth environment, but it can provide references for further analysis of the relationship between them.

Historical climate change has shaped the current distribution pattern of species (Xiao et al. 2021), while the current distribution pattern of species reflects the characteristics of historical climate change (Zhang et al. 2022). It is predicted that the suitable areas of ratoon rice in China is mainly distributed in the south of Qinling–Huaihe Line (92.39–121.96E, 18.23–30.36 N) under the current climate scenario, with a northern boundary of 30 ± 1 N. The total area of suitable areas is 193.90 × 10^4^ km^2^, of which the highly‐suitable areas (103.22–117.09E, 26.39–29.01 N) is 28.02 × 10^4^ km^2^. Among the highly‐suitable areas, Sichuan, Hunan, Hubei and Chongqing account for 25.02%, 16.38%, 14.38% and 8.71% respectively, ranking first in China, which is consistent with the actual distribution pattern of ratoon rice in China (Li et al. 2009; Song et al. 2020). The study further find that the potential suitable area of ratoon rice will only increase by 0.5% to 4.1% under future scenario, while the area of highly‐suitable areas will increase by 35.3% to 80.7%, and there is a significant trend to the northeast. In view of this, we speculate that in the future, the autumn thermal conditions north of the Qinling–Huaihe Line will remain within the range that restricts the northward expansion of ratoon rice. In contrast, moderately increased autumn thermal conditions south of the Qinling–Huaihe Line will be favorable for the cultivation of ratoon rice. Most of the currently moderately and marginally suitable areas for ratoon rice in the middle and lower reaches of the Yangtze River are expected to shift into highly and moderately suitable zones. In particular, new highly suitable areas will emerge in southern Hunan, the central parts of Hubei, Jiangxi, and Anhui, and western Zhejiang, where appropriately scaled cultivation could be prioritized in the future.

There are certain differences in the predictive performance among different niche models (Zhu et al. 2014). Studies have shown (Wang et al. 2007) that, compared with other niche models, the MaxEnt model is characterized by good stability and high prediction accuracy. In addition, this model can achieve reliable prediction results even with a relatively small number of occurrence records (Chen et al. 2012), making it one of the most widely applied species distribution models at present. However, since MaxEnt relies solely on presence data, if background points or pseudo‐absence points are not properly set, it may overestimate the potential distribution range of species (Liu et al. 2022). Currently, various models based on different algorithms, such as GARP, MARS, DOMAIN, BIOCLIM, CLIMEX, and MaxEnt, have been applied in habitat suitability prediction. Therefore, future studies should incorporate multiple models to improve prediction accuracy and provide more precise theoretical and technical support for predicting the suitable areas of ratoon rice.

CMIP6 includes multiple global climate models, which differ substantially in terms of physical process parameterization, spatial resolution, and the coupling of Earth system components, leading to varying simulation capabilities for climate variables. At the same time, emission scenarios depend on socioeconomic assumptions such as future population and energy structures; however, actual policy adjustments (e.g., the “dual‐carbon” targets) or technological breakthroughs may deviate from the predefined pathways, resulting in projection biases. In this study, the BCC‐CSM2‐MR global climate model from CMIP6 was employed to predict changes in the suitable areas of ratoon rice under three future scenarios. The results showed that by the 2090s, the total suitable area of ratoon rice would reach its maximum under the SSP2‐4.5 scenario, the highly suitable area would peak under the SSP5‐8.5 scenario, and the moderately and marginally suitable areas would peak under the SSP1‐2.6 scenario. These findings indicate that climate change exerts uncertain effects on the potential distribution of ratoon rice under different emission scenarios. In future research, multi‐model ensemble simulations will be required to reduce the projection bias associated with any single model.

Although the study made a good prediction for the suitable areas of ratoon rice in China, there are still some problems. For example, the study only predicted the potential suitable areas of ratoon rice based on climate data, but Song et al. (2020) found that soil nutrients, cultivation techniques and other factors also had significant effects on the distribution of ratoon rice. In addition to the influence of climate and altitude factors, we will also consider the reliable expression of other comprehensive factors in future studies. At the same time, we will use a variety of evaluation indicators to verify the accuracy of the species distribution prediction model, select a more reasonable prediction model and prediction method, and provide more accurate theoretical basis and technical support for the prediction of the suitable area of regenerated rice.

## 5 Conclusion

Based on the MaxEnt model and 167 occurrence records of ratoon rice, the accumulated temperature during the safe growth period (T), the number of safe growth days (Day), altitude (Alt), the hydrothermal coefficient in August (K_8_), the mean temperature in September (Tav_9_), and precipitation from March to September (Prec_39_) were identified as the key environmental variables influencing the potential distribution of ratoon rice. Among them, the accumulated temperature during the safe growth period and the number of safe growth days jointly constrained the expansion of suitable areas beyond the Qinling–Huaihe Line, serving as two crucial environmental factors responsible for the concentration of highly suitable areas between 26° N and 29° N. Under climate change scenarios, the potential suitable distribution of ratoon rice remains primarily south of the Qinling–Huaihe Line, without a marked northward shift, with the northern boundary at approximately 30° ± 1° N. The total suitable area is projected to increase by 5.5%–11.95%. Highly suitable areas are mainly distributed in the southern Sichuan Basin, western Chongqing, and the middle and lower reaches of the Yangtze River, showing a northeastward expansion trend. The area of highly suitable zones is expected to increase substantially by 35.3%–80.7%, with new expansion concentrated in southern Hunan, the central parts of Hubei, Jiangxi and Anhui, and western Zhejiang. Therefore, relevant authorities should pay particular attention to the middle and lower reaches of the Yangtze River, where the introduction, cultivation, and appropriately scaled planting of ratoon rice can be prioritized.

## Author Contributions

**Wei Luo:** investigation (equal), project administration (equal), software (equal), writing – original draft (lead). **Lixin Yuan:** software (equal), validation (equal), visualization (equal), writing – original draft (equal). **Shengmin Yan:** data curation (equal), investigation (equal), validation (equal), visualization (equal). **Linlin Wang:** investigation (equal), resources (equal), software (equal). **Yongfu Yu:** investigation (equal), resources (equal), software (equal). **Huan Deng:** data curation (equal), investigation (equal). **Jinpeng Zhao:** conceptualization (equal), formal analysis (lead), methodology (lead), validation (equal), writing – review and editing (lead). **Rulin Wang:** conceptualization (equal), formal analysis (equal), methodology (equal), validation (equal), writing – review and editing (equal).

## Funding

This work was supported by the National Key Research and Development Program of China (2024YFD2301305), the Key Innovation Team of Sichuan Provincial Meteorological Service (SCQXZDCXTD202403), the National Natural Science Foundation of China (U20A2022‐1), the Heavy Rain and Drought‐Flood Disasters in Plateau and Basin Key Laboratory of Sichuan Province (SCQXKJYJXZD202408; SCQXKJYJXMS202411; SCQXKJYJXZD202509), and the China Meteorological Administration Innovation and Development Special Project (CXFZ2025J053).

## Conflicts of Interest

The authors declare no conflicts of interest.

## Data Availability

The data supporting the results are available in a public repository at: https://doi.org/10.6084/m9.figshare.30364189.v1.
